# HOXD9 promote epithelial‐mesenchymal transition and metastasis in colorectal carcinoma

**DOI:** 10.1002/cam4.2967

**Published:** 2020-04-12

**Authors:** Mengwei Liu, Yizhi Xiao, Weimei Tang, Jiaying Li, Linjie Hong, Weiyu Dai, Wenjing Zhang, Ying Peng, Xiaosheng Wu, Jing Wang, Yaying Chen, Yang Bai, Jianjiao Lin, Qiong Yang, Yusi Wang, Zhizhao Lin, Side Liu, Jing Xiong, Jide Wang, Li Xiang

**Affiliations:** ^1^ Guangdong Provincial Key Laboratory of Gastroenterology Department of Gastroenterology Nanfang Hospital Southern Medical University Guangzhou China; ^2^ Department of Medical Oncology The First people's Hospital of Yunnan Province Medical School of Kunming University of Science and Technology Kunming China; ^3^ Department of Gastroenterology The Third Affiliated Hospital of Guangzhou Medical University Guangzhou China; ^4^ Department of Gastroenterology Longgang District Peopl Hospital Shenzhen China; ^5^ The Second Affiliated Hospital of University of South China Hengyang China

**Keywords:** colorectal cancer, epithelial‐mesenchymal transition, HOXD9, metastasis, TGF‐β1

## Abstract

**Background:**

HOXD9, a Hox family member, is involved in cancer growth and metastasis. But, its regulation mechanism at the molecular level particularly in colorectal cancer (CRC), is mostly unknown.

**Methods:**

The HOXD9 protein expression levels were analyzed using immunofluorescence, immunohistochemistry (IHC) assays, and western blot. The in vivo and in vitro roles of HOXD9 in CRC were determined using colony formation and EdU incorporation, CCK‐8, wound scratch and transwell invasion assay, and animal models.

**Results:**

Expression of HOXD9 was higher in CRC than in matched healthy tissues. High expression of HOXD9 has significantly associated with the American Joint Committee on Cancer (AJCC) stages, tumor differentiation, lymph node metastasis, and other serious invasions, and it had a poor prognosis. In vitro, HOXD9 encouraged proliferation, movement and EMT processes in cells of CRC. Also, TGF‐β1 promoted the expression of HOXD9 and this effect was dependent on the dose and downregulation of HOXD9 repressed TGF‐β1 ‐induced EMT. In vivo, HOXD9 promoted the invasive and metastasis of CRC cells via orthotopic implantation.

**Conclusions:**

The ectopic expression of HOXD9 promoted the invasion metastasis in cells of the colorectal tumor by induction of EMT in vitro and vivo.

## Graphical abstract

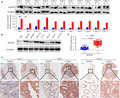

**Abbreviations**
AJCC: American Joint Committee on Cancer
CRC: colorectal cancer
EMT: epithelial‐mesenchymal transition
HOX: homeobox
IHC: immunohistochemical staining
RT‐PCR: reverse transcriptase polymerase chain reaction
TMA: tissue microarray

## 1 INTRODUCTION

Colorectal cancer (CRC) has been a public health concern for years, and it accounts for approximately 9% of all cancer incidence rates, this represents more than 1.4 million new cases yearly.^1^ The disease is the third most commonly occurring malignant tumor in the world. It is the fourth most frequent cause of mortality with an oncological origin. Although several treatment options are available, such as a blend of surgery, chemotherapy, and radiation therapy, close to 50% of the cases progress to recurrent and distant disease. Thus, it is very crucial to investigate the molecular mechanisms at play in CRC development, which may probably lead to new and practical antitumor approaches for patients with metastatic CRC.

The HOX genes are classified under the superfamily of homeobox genes, which encode for transcription factors with crucial roles in development.^2^, ^3^ The HOX genes comprise a conserved 183 bp sequence and encode nuclear proteins known as homeoproteins. The HOX proteins regulate various cellular processes by controlling the expression of several downstream target genes.^4^, ^5^ Hence, it is common for a single homeoprotein to cause pleiotropic effects on cell behavior, such as alterations in survival, invasion, proliferation, and migration.

The HOX genes belong to a family of 39 transcription factors. These factors are further separated into groups, which include HOXA, HOXB, HOXC, and HOXD.^6^, ^7^, ^8^, ^9^ The groups are found occurring in tandem on separate chromosomes. The HOXD9 gene is an example of HOXD genes that are located close to the 3′ end of a chromosome. It plays a role in the development and progression of cancer.^10^, ^11^ Studies have suggested that HOXD9 is highly expressed in most squamous cell carcinomas of the human esophagus. It assists in cell survival and proliferation. It has been recently shown that HOXD9 encourages epithelial‐mesenchymal transition (EMT) and the spread of cancer by regulation of ZEB1.^10^ In contrast, knockdown of ZEB1 hinders the induction of HOXD9 invasion and EMT in carcinoma cells of the liver.^11^ Moreover, HOXD9 has been implicated as a tumor‐inducing factor in gastric cancer.^12^ But, the function of HOXD9 genes in the development and growth of CRC is yet to be revealed.

The current study suggested that increased expression of HOXD9 is associated with proliferation, invasion, and spread of the tumor and indicates poor prognosis in patients suffering from CRC. Moreover, ectopic expression of HOXD9 improved EMT and was induced by TGF‐β. Taken together, our results reported for the first time that high expression of HOXD9 could enhance the formation of aggressive characteristics of colorectal tumor cells.

## 2 MATERIALS AND METHODS

### 2.1 Cell culture and reagents

A non‐tumourigenic immortalized adult human colon epithelial cell line, FHC, was obtained from the ATCC. The colon cancer cell lines SW620, LoVo, SW1116, SW480, HT‐29, DLD1, and LS174T cells were grown in RPMI 1640 containing 10% fetal bovine serum (GIBCO), 1% glutamine (Life Technologies), penicillin G (100 µ/mL) and streptomycin (100 μg/mL) (Sigma‐Aldrich). All the cell cultures were maintained as a monolayer culture at 37°C in a humidified atmosphere containing 5% CO_2_.

Recombinant human TGF‐1 (240‐B) and monoclonal anti‐TGF‐β1 antibody was purchased from R&D Systems. Rabbit antibodies against HOXD9 (SAB4200029‐200UL), E‐cadherin (20874‐1‐AP), AKT (9272S) and ErK1/2 (#4695S), or mouse antibodies against CDK4 (#2906S) and CDK6 (#3136S) were purchased from Cell Signaling. Mouse antibody against vimentin (60330‐1‐lg) was purchased from proteintech. Mice antibodies against Cyclin B1 (sc‐245), Cyclin D1 (sc‐8396), MMP‐9 (sc‐21733) were purchased from Santa Cruz Biotechnology. Mouse antibody against β‐tubulin was purchased from Ray Antibody Biotech. Alexa Fluor ®488—Conjugated Goat anti‐ Rabbit IgG (conjugated to red fluorescent dyes, ZF‐0511)and Alexa Fluor ®488—Conjugated Goat anti‐Mouse IgG (conjugated to green fluorescent dyes, ZF‐0512) were purchased from ZSGB‐BIO, China.

### 2.2 Tissue microarray (TMA) and immunohistochemical (IHC) analysis

Tissue microarray (TMA) CRC tissues of human and matched colonic noncancerous tissues were procured from Superchip (NO. HColA180Su10, Shanghai, China). In the current study, all tissues (100 in number) were adenocarcinomas derived from 100 patients with CRC who underwent surgery between April 2008 and December 2008. All cases were surgically removed and confirmed by pathological examination. The HOXD9 expression was spotted using IHC in TMA slides. Tumor staging was defined by following the standards for histological classification as outlined by the International Union against Cancer (UICC). In the IHC assay, paraffin‐embedded, formalin‐fixed, sections of the tissues (5 μm) were cleaned off paraffin using xylene and dried using gradient concentrations of molecular grade ethanol. The endogenous activity of peroxidase was stopped (0.35% H_2_O_2_ in PBS buffer), and antigens were recovered by warming utilizing a microwave (350 W). Blocking of nonspecific binding was achieved by 1% bovine serum albumin dissolved in PBS buffer. The Sections were incubated together with primary antibodies then incubated with peroxidase‐conjugated anti‐rabbit secondary antibody (Dako) (1:100). To visualize expression levels of HOXD9, 1 mg/mL 3, 3'‐diaminobenzidine counterstained using hematoxylin was used. Independent scoring of tissue slides was conducted by two investigators. The cancer cell staining intensity was scored as zero (negative), one (light yellow, weak expression), two (yellow, moderate expression), and three (yellowish‐brown, strong expression). A score of ≥2 and at least 50% of protein‐positive cells were regarded as high expression/overexpression and a score of <2 with <50% of protein‐positive cells was considered low expression. This study was authorized by the human ethics committees of the applicable institutions.

### 2.3 DNA constructs

The RT‐PCR method was used to obtain complementary DNA (cDNA) of a healthy human, which corresponded to the complete‐length of HOXD9 gene. The products obtained after PCR were subcloned into the pENTER‐FLAG mammalian expression vector (ViGene Biosciences). A previously described method which involves using the same selection process and plasmid was used to obtain populations of pENTER and pENTER HOXD9 stable transfectants.^13^

### 2.4 Cell cycle analysis

The HOXD9 shRNA/scramble shRNA infected cells were gathered and rinsed two times using ice‐cold phosphate‐buffered saline (PBS), and then fixed using ice‐cold ethanol (70%). The cells were left to stand overnight in the fixative. They were subsequently dried using PBS at 4°C for 30 minutes, and samples were then stained in the dark for 30 minutes using 400 µg/mL propidium iodide (Sigma‐Aldrich) comprising of 125 µ/mL protease‐free RNase, a flow cytometer (FACS Calibur, Becton Dickinson) was used to analyze the samples. Analysis of the cell cycle was conducted with FlowJo software.

### 2.5 RT‐ PCR analysis

Colorectal mucosa tissues, including cell lines, were used for total RNA extraction using Trizol reagent kit (Invitrogen, USA). Total RNA (1 μg) reverse transcription of RNA to cDNA was achieved by using the PrimeScript™ II kit (Gibco BRL) as outlined in the manufacturer's guidelines.

Applied Biosystems Sequence Detection System 7900 (ABI Prism 7900HT, Applied Biosystems Company, USA) was used to perform qPCR. The reaction included a 10 µL mix composed of Power SYBR GREEN PCR Master Mix (Applied Biosystems, Foster City, CA, USA), 300 ng of cDNA template, and 500 nmol of both forward and reverse primer. The following PCR conditions were used: Initial denaturation for 5 minutes at 94°C; and 30 rounds of denaturation at 94°C for 30 seconds, annealing at 55°C for 30 seconds, and elongation at 72°C for 1 minute. In each qRT PCR performed, each cDNA sample was prepared in duplicate, and the mean relative fold mRNA expression levels were evaluated with the 2^−ΔΔC^_t_technique using GAPDH as the internal control.

The following are the primer sequences for RT‐PCR: E‐cadherin forward 5'‐TGCCCAGAAAATGAAAAAGG‐3' and reverse 5'‐GTGTATGTGGCAATGCGTTC‐3' (200 bp); GAPDH forward 5'‐GTCAACGGATTTGGTCGTATTG‐3' and reverse 5'‐CTCCTGGAAGATGGTGATGGG‐3' (204 bp).

### 2.6 Western blot analysis

To conduct western blot analysis, cancer tissue which was previously frozen (30 mg), treated cells, or adjacent healthy tissues were lysed using RIPA lysis buffer comprising of 1% protease inhibitor cocktail (Cwbiotech). The same quantities of protein (approximately 30 μg) were resolved by gel electrophoresis (SDS‐PAGE) and relocated onto a polyvinylidene fluoride (PVDF) membrane. The PVDF membrane was then incubated with primary antibodies after blocking using skimmed milk (5%). The membrane was washed thrice with Tris‐buffered saline comprising of tween 20, and the membrane was subsequently incubated at room temperature with horseradish peroxidase‐conjugated secondary antibody for one hour. Visualization of the protein bands was achieved using an ECL detection kit (Millipore, Billerica, MA).

### 2.7 Colony‐forming and cell proliferation assays

To evaluate cell proliferation, cancerous cells were seeded at 5 × 10^3^ cells per well in plates having 96‐wells and then incubated for three days at 37°C. Subsequently, an aliquot (10 μL) of Cell Counting Kit‐8 (CCK‐8) reagent (Dojindo, Japan) was pipetted to the cells. After three hours of incubation, the spectrophotometer (Bio‐Rad, USA) was used to measure the absorbance at 450 nm. To conduct the colony‐forming assay, plating of four transfectant cell lines was performed at 100 cells/wells in plates having 6‐wells. The plates were subsequently incubated for 14 days in RPMI‐1640. After the incubation, the plates were stained with crystal violet. Colonies were counted from the images of the stained plates after they were captured. Every treatment was conducted thrice.

### 2.8 EdU incorporation assay

Six‐well dishes were used to seed the cells of CRC at a density of 1 × 10^5^ cells/mL. The cells were allowed to stick for 12 hours, after which they were incubated for four hours with 5‐ethynyl‐2’‐deoxyuridine (EdU) before detection. The Cell‐Light EdU Cell Proliferation Detection kit (RiboBio) was used to evaluate the rate of cell proliferation according to the guidelines of the manufacturer.

### 2.9 Invasion and wound scratch assays

The wound scratch assay and trans well chamber assay was used to test the cell migration and invasion abilities. Assay for wound healing was conducted to detect the cell movement. Six‐well plates were used to culture the transfectant cells until confluent. An aseptic tip was used to injure the cell layer. The cells were treated with 10 μg/mL of mitomycin C during the culture period to inhibit cell proliferation, and this was done after 48 to 72 hours of incubation. Photomicrographs were taken using a phase‐contrast microscope. Each experiment was performed thrice.

Trans well chamber culture system (Becton Dickinson) was used to detect the migratory ability of the CRC cells. The cells were moved into the upper chamber of the wells having growth medium devoid of serum (1 × 10^5^ cells/well of 24‐well 8 mm transwell) after 48 hours of incubation at 37°. A chemoattractant comprising of growth medium with FBS (10%) was incorporated into the lower chamber. After 24 hours of incubation at 37°, cotton swabs were used to move the cells (nonmigratory cells), which were in the upper surface of the upper chamber. The cells on the lower surface were fixed using a fixative, followed by staining using the Giemsa stain. A light microscope (Leica, Germany) was used to count the number of cells invaded.

### 2.10 Immunofluorescence

Coverslips were seeded with cells followed by 24‐36 hours of culture. The PBS was used to rinse cells before they were fixed using PFA (4%) at room temperature for 15 minutes. Subsequently, 1% Triton X‐100/1% bovine serum albumin in PBS was used to induce permeabilization of the cells. Cells were incubated with the primary antibodies (anti‐E‐cadherin and anti‐vimentin) for 4 hours, then incubated with Texas Red (TR, excitation wavelengths: 589 nm)‐conjugated or fluorescein isothiocyanate (FITC, excitation wavelengths: 488 nm)‐conjugated secondary antibodies and left to stand for 1 hour at room temperature. The immunofluorescence complexes were visualized with an Olympus (FV1000 Spectral) microscope.

To determine the F‐actin cytoskeleton, CRC cells were cultured on glass coverslips for 24 hours. Cells were subsequently permeabilized and fixed as earlier described; this was followed by staining using FITC‐conjugated phalloidin at 1:50 in PBS. Nuclei were stained using 1 µg/mL Hoechst 22 358, and cells were observed with a fluorescence microscope.

### 2.11 Construction of lentivirus

Genechem (Shanghai, China) was used to construct lentivirus expressing HOXD9 (LV‐HOXD9)/EGFP by employing Ubi‐MCS‐3FLAG‐CBh‐gcGFP‐IRES‐puromycin vector. The Ubi‐MCS‐3FLAG‐CBh‐gcGFP‐IRES‐puromycin empty vectors (Shanghai Genechem Co. Ltd, China) served as controls. Double‐stranded oligonucleotides encoding human HOXD9‐vshRNA: (NM_014213: CCGGCAGCAACTTGACCCAAACATCAAGAGTGTTTGGGTCAAGTTGCTGTTTTTG). Selected pools of overexpressing and knockdown cells were used to conduct succeeding experiments.

### 2.12 In vivo metastatic model and bioluminescent imaging

Mice (Female nude) [BALB/c nu/nu], which were 4‐6 weeks old, were individually kept in ventilated enclosures under aseptic conditions as recommended by the institutional guidelines for animal care. All mice experiments in the current study were authorized by the Committee on the Use of Live Animals in Teaching and Research, Southern Medical University (Guangdong, China). A suspension consisting of single cells of 5 × 10^6^ LoVo/EGFP/vector or LoVo/EGFP/HOXD9 ‐transduced cells in 100 μL of PBS was inoculated into the cecal wall as described in literature.^14^ The growth of the cancer cell was checked after 45 days using bioluminescent imaging (BLI) with the IVIS100 Imaging System (Kodak, Rochester, NY, USA). Mice were sacrificed, and analysis of the metastatic tissues was conducted by qRT‐PCR and hematoxylin and eosin staining, IHC.

### 2.13 Statistical analysis

SPSS software (version 20.0 PSS) was used for data analysis and presentation. And numerical data were presented as mean ± SD. Logistic regression was conducted. Kaplan‐Meier and log‐rank tests were used to analyze survival rates. Quantitative data were analyzed with Student's *t *test (two‐tailed) and *P* < .05 was considered statistically significant.

## 3 RESULTS

### 3.1 Upregulation of HOXD9 protein in CRC tissues

**FIGURE 1:**
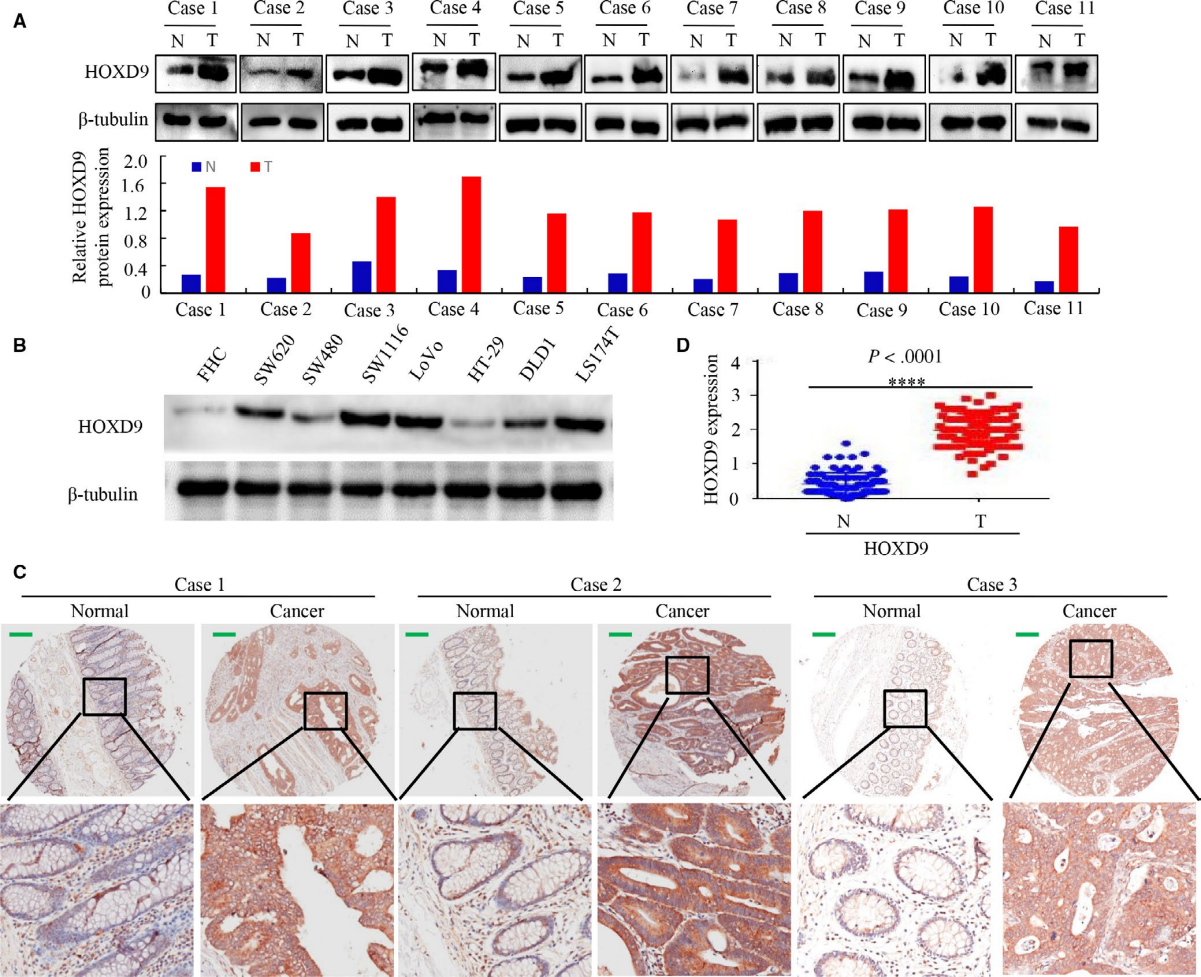
HOXD9 expression in colorectal cancer (CRC) is higher compared to healthy cells. A, Western blot results of HOXD9 protein levels in 11 CRC tissues. Comparative levels of protein expression were measured by evaluating the greyscale of each band with Quantity One Software. B, Whole lysates of SW620, FHC, SW1116, SW480, DLD1,LoVo, LS174T, and HT29 cells were gathered, and HOXD9 was detected by western blot using b‐tubulin as the internal control. C, HOXD9 expression in CRC and healthy tissues was evaluated using a TMA. D, Semiquantitative scoring of proteins (HOXD9) in the cancerous colon and healthy tissues. ****P < .001 between cancer and healthy tissues. Scale bars in C represent 25 μm

We previously reported that HOXD9 is highly expressed in GC,^12^ we determined whether HOXD9 has any effect on GC proliferation, invasion, and metastasis. Firstly, we analyzed HOXD9 levels in fresh CRC samples. The result showed that, relative to paired adjacent noncancerous tissues, HOXD9 was markedly increased in the eleven tumor specimen (Figure 1A). Secondly, HOXD9 levels were examined in CRC cell lines. Figure 1B shows that HOXD9 protein was remarkably highly expressed in the CRC cell compared to the healthy human colon cell line (FHC).

Thirdly, we have performed to examine the protein level of HOXD9 inpatient using high throughput tissue microarray immunohistochemistry (TMA‐IHC). The results revealed that HOXD9 was markedly upregulated in CRC tissues but was only marginally detectable in healthy colon tissues, as exemplified in Figure 1C. Moreover, Cancer tissues showed higher HOXD9 expression relative to adjacent healthy colon mucosa samples by semiquantitative scoring (Figure 1D).

**FIGURE 2:**
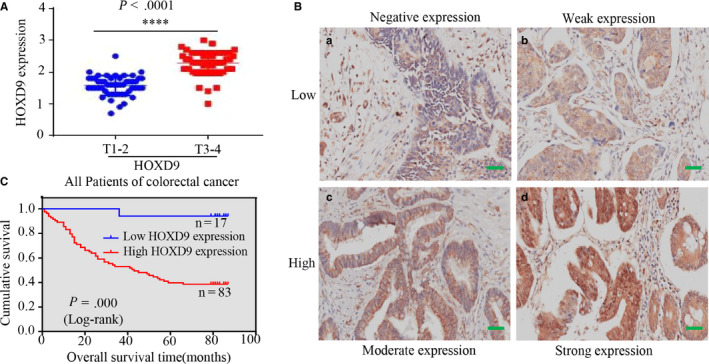
HOXD9 was higher in advanced stages and associated with poor prognosis. A, In cancerous tissues, the level of HOXD9 in advanced (stages 3‐4) colorectal cancer (CRC) was higher compared to that in early (stages I‐II) CRC; ****P < .001. B, The HOXD9 protein expression analysis of CRC by IHC. Immunoreactivity in staining of HOXD9 was confined in cells as follows: (a) No expression; (b) mild expression; (c) average expression; and (d) high expression in CRC. C, Kaplan‐Meier survival analysis of overall survival in all patients according to the expression of HOXD9. *P* values were calculated by the log‐rank test. Hazard ratio = 3.39, 95% CI = 1.77‐6.493. Scale bars in B represent 25 μm

According to the median expression level, HOXD9 was grouped into low and high expression groups, and these groups were used to determine the relationship between HOXD9 and the clinical characteristics and prognosis. Statistical analyses revealed that HOXD9 levels were not related to the age (< 60 years vs. >60, *P* = .115), gender (*P* = .788) or tumor size (<5 cm vs. ≧5 cm, *P* = .592) but were highly related to lymph node metastasis (*P* = .034), differentiation (*P* = .000), AJCC stage (I/II vs. III/IV, *P* = .032), and extra serious invasion (*P* = .020) (Table 1). Furthermore, HOXD9 expression was higher in patients with stage T3‐T4 stage than those with stage T1‐T2 (Figure 2A).

**TABLE 1:** Correlation between HOXD9 protein expression and the clinicopathological parameters of colorectal cancer (CRC)

Characteristics	Case	HOXD9 expression (%)		*P* values
		Low expression	High expression	
Age (years)				
<60	24	7 (29.2%)	17 (70.8%)	.007
≥60	76	10 (13.2%)	66 (86.8%)	
Gender				
Male	59	11 (18.6%)	48 (81.4%)	.798
Female	41	6 (14.6%)	35 (85.4%)	
Differentiation				
Well	17	6 (35.3%)	11 (64.7%)	.386
Moderate	74	10 (13.5%)	64 (86.5%)	
Poor	9	1 (11.1%)	8 (88.9%)	
AJCC stage				
I/II	51	13 (25.5%)	38 (74.5%)	.000
III/IV	49	4 (8.2%)	45 (91.8%)	
Lymph node metastasis				
No	52	13 (25%)	39 (75%)	.023
Yes	48	4 (8.3%)	44 (91.7%)	
Tumor size				
<5 cm	36	5 (13.9%)	31 (86.1%)	.163
≥5 cm	64	12 (18.8%)	52 (81.2%)	
Extra serous invasion				
No	71	16 (22.5%)	55 (77.5%)	.024
Yes	29	1 (3.4%)	28 (96.6%)	

Furthermore, the log‐rank test and Kaplan‐Meier survival analyses confirmed that high expression of HOXD9 was strongly related to shortened overall patient survival based on the TMA data (Figure 2B and C).

Together, our dataset indicated that increased HOXD9 levels are positively related to poor outcome, metastasis, and progression in patients with CRC.

### 3.2 HOXD9 enhances CRC cell proliferation

**FIGURE 3:**
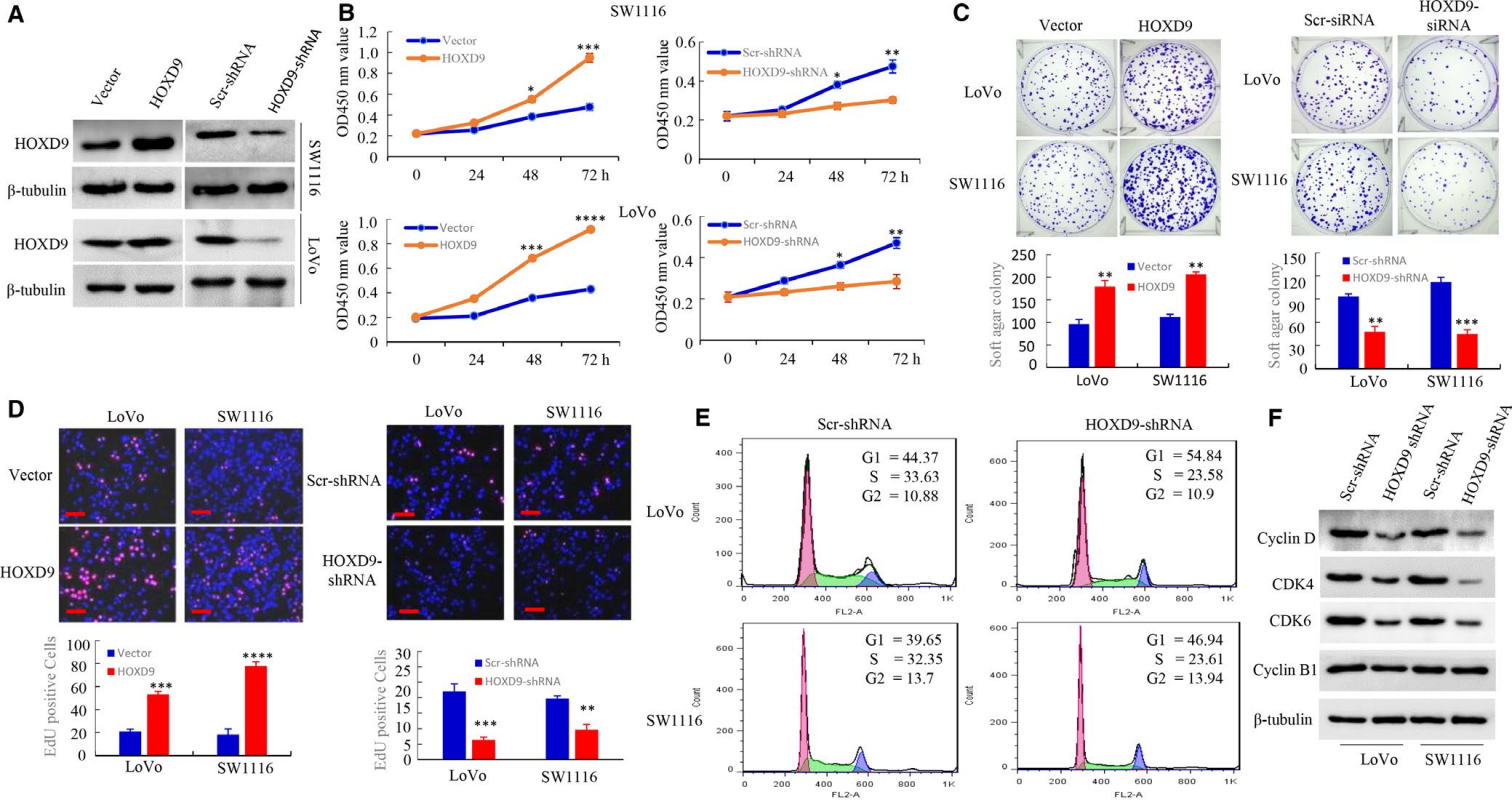
The expression of HOXD9 controls the cell cycle and proliferation of colorectal cancer (CRC) cells in humans. A, Vector, HOXD9, HOXD9 shRNA or scrambled (Scr) shRNA CRC cells were analyzed using Western blot, and b‐tubulin was used as the internal control. B, Cells seeded in triplicate in plates (96‐well) were gathered 24, 48‐ and 72‐h post‐seeding and analyzed using the CCK‐8 assay. (n = 3); *P > .05; **P < .05; ***P < .01 and ****P < .001. C, Cells were plated in dishes (used for tissue culture) with full culture medium. Two weeks after plating, the cell colonies stained using 0.005% crystal violet and visualized. *P < .05; ***P < .01. D, DNA synthesis by the cells of CRC was determined by using an EdU incorporation assay following the indicated transfections at 48 hours. ***P < .01 and ****P < .001, vector vs. HOXD9; **P < .05 and ***P < .01, Scr shRNA vs. HOXD9 shRNA. E, Analysis of the cell cycle was examined in LoVo and SW1116 cells using FACScan. RNAi‐mediated suppression of HOXD9 expression controlled the checkpoint of G0/G1. F, Preparation of whole‐cell lysates of parental cells of CRC was performed, and expression of the protein was detected using the western blot technique. The pictures represent three different experiments with similar results. scale bars in D represent 100 μm

To investigate the mechanism by which HOXD6 contributes to colorectal cancer progression, we constructed stable transfectants using HOXD9‐sense plasmids or knockdown of HOXD9 with shRNA in LoVo and SW1116 cells and was confirmed by western blot analysis (Figure 3A). To explore the function of HOXD9 on cell growth, CCK‐8, colony formation, and EdU incorporation assays were performed to evaluate the influence on proliferation. The CCK‐8, showed that HOXD9 overexpression accelerated spread compared to the control group. In contrast, down‐regulated HOXD9 showed the opposite result (Figure 3B). Figure 3C shows that HOXD9 promoted colony formation ability but that HOXD9 knockdown inhibited CRC cell growth.

Moreover, the EdU incorporation assay revealed distinct differences in the proliferation of CRC cell lines (Figure 3D). Also, cell cycle profiles were evaluated by FACS analysis. The knock‐down of HOXD9 increased the number of cells entering G0‐G1 phase, accompanied with a reduction in the quantity of cells at S phase relative to cells transfected with an empty vector (Figure 3E).

We subsequently assessed the expression of proteins associated with cell proliferation. This revealed that the protein expression of CDK4, CDK6, and Cyclin D was suppressed by knockdown of HOXD9. However, the quantities of Cyclin B1 were not altered following HOXD9 knock‐down (Figure 3F). These results suggested that HOXD9 is involved in mediating the transition of cells through the G1‐phase.

Overall, these findings show that HOXD9 has pivotal roles in promoting proliferation in CRC cells in vitro.

### 3.3 HOXD9 promotes CRC cell migration and invasion through enhancing EMT

**FIGURE 4:**
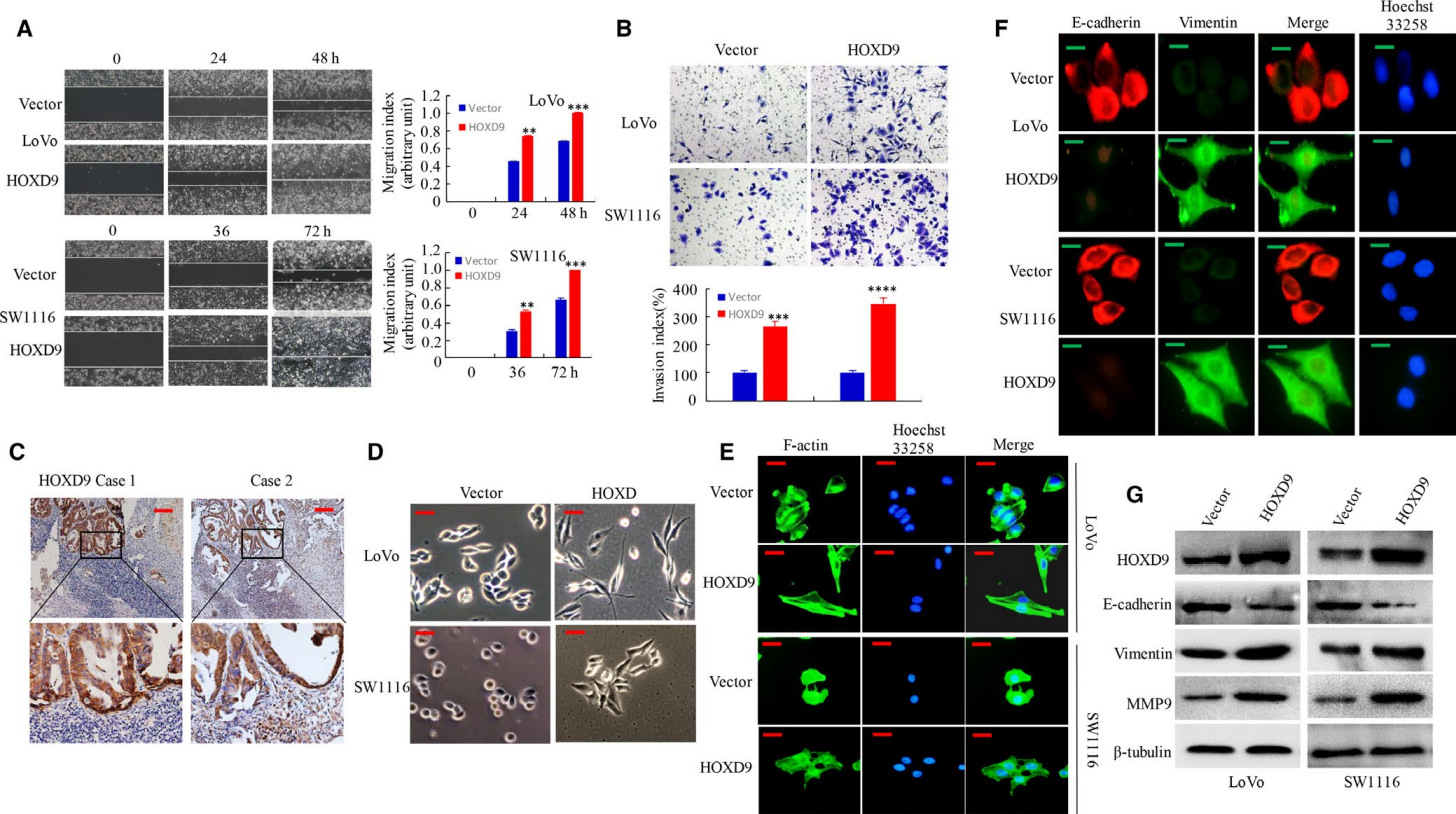
HOXD9 enhances the migration and invasion of colorectal cancer (CRC) cells by promoting the EMT process. A, The migration of CRC cells with high expression of HOXD9 was evaluated using a wound‐healing assay. **P < .05 and ***P < .01, vector vs. HOXD9. B, Cell invasion of CRC cells showing high expression of HOXD9 was evaluated using a Matrigel invasion chamber. The invaded cells of CRC were recorded. ***P < .01 and ****P < .001, vector vs. HOXD9. C, The results of IHC staining for detection of HOXD9 in CRC tissues and lymph node metastatic tumor tissues. D, Morphology of expression levels of HOXD9 in vector or HOXD9 cells as visualized by the use of phase‐contrast microscopy. E, Vector or HOXD9 stable transfectants were stained using rhodamine‐phallotoxin, and F‐actin filaments were visualized using fluorescence microscopy. F, IF staining of E‐cadherin and vimentin in the indicated CRC cells. G, The EMT biomarkers expression which included vimentin, E‐cadherin, and MMP9, was detected using western blotting at 48 h post‐transfection. Scale bars represent 100 μm in C; 50 μm in D and E; 20 μm in F

Enhances cell invasion and migration ability are up‐regulated in tumors with high metastatic activity. Thus, the impact of forced upregulation of HOXD9 on the migration and invasion of CRC tumors was examined. Results revealed that HOXD9 upregulation in CRC increased the migration ability by wound scratch assay. The migration index of the ectopic expression of HOXD9 cells was increased by 45% and 38% at 24 and 48 in LoVo cells, by 48% and 27% in SW1116 cells, respectively (Figure 4A). Moreover, overexpression of HOXD9 in CRC cells enhanced the number of invaded cells by transwell invasion assay. After HOXD9 over‐expression, the invasiveness of CRC cells was decreased by 61.5% and 70.6% as compared with the vector cells (Figure 4B). Furthermore, we measured the level of HOXD9 metastatic cells of lymph nodes with IHC. The HOXD9 protein was highly expressed in metastatic lymph nodes tissues of CRC from 21/23 patients, as exemplified in two patients (Figure 4C).

In human cancer, the activation of EMT process relates to worse prognosis and advanced disease.^15^ Therefore, the effect of ectopic HOXD9 in the EMT process of CRC cells was investigated. Results showed that forced expression of HOXD9 acquired a spindle‐shaped fibroblast‐like appearance and generally lost intercellular connectivity, a key feature of EMT (Figure 4D). Because previous studies have suggested that remodeling of actin regulates EMT in metastasizing tumor cells,^16^ the F‐actin cytoskeleton was visualized by phalloidin staining. Figure 4E, transfection of CRC cells with HOXD9 led to a fibroblastic spindle‐like morphology, and HOXD9 was showed uniform distribution at the rim zone of the protrusion and in cytosol compared to cells transfected with empty vector. Ectopic overexpression of HOXD9 in LoVo and SW1116 cells also resulted in higher vimentin levels but lower E‐cadherin levels based on immunofluorescent assay (Figure 4F). The changes in the EMT process were evidenced by the transition epithelial biomarkers (E‐cadherin) to mesenchymal biomarkers (MMP9 and vimentin) by Western blot analysis (Figure 4G).

Together, the results described reveal that overexpression of HOXD9 induces EMT and facilitates CRC cell invasion.

### 3.4 HOXD9 mediates TGF‐β‐triggered EMT and invasion in CRC cells

**FIGURE 5:**
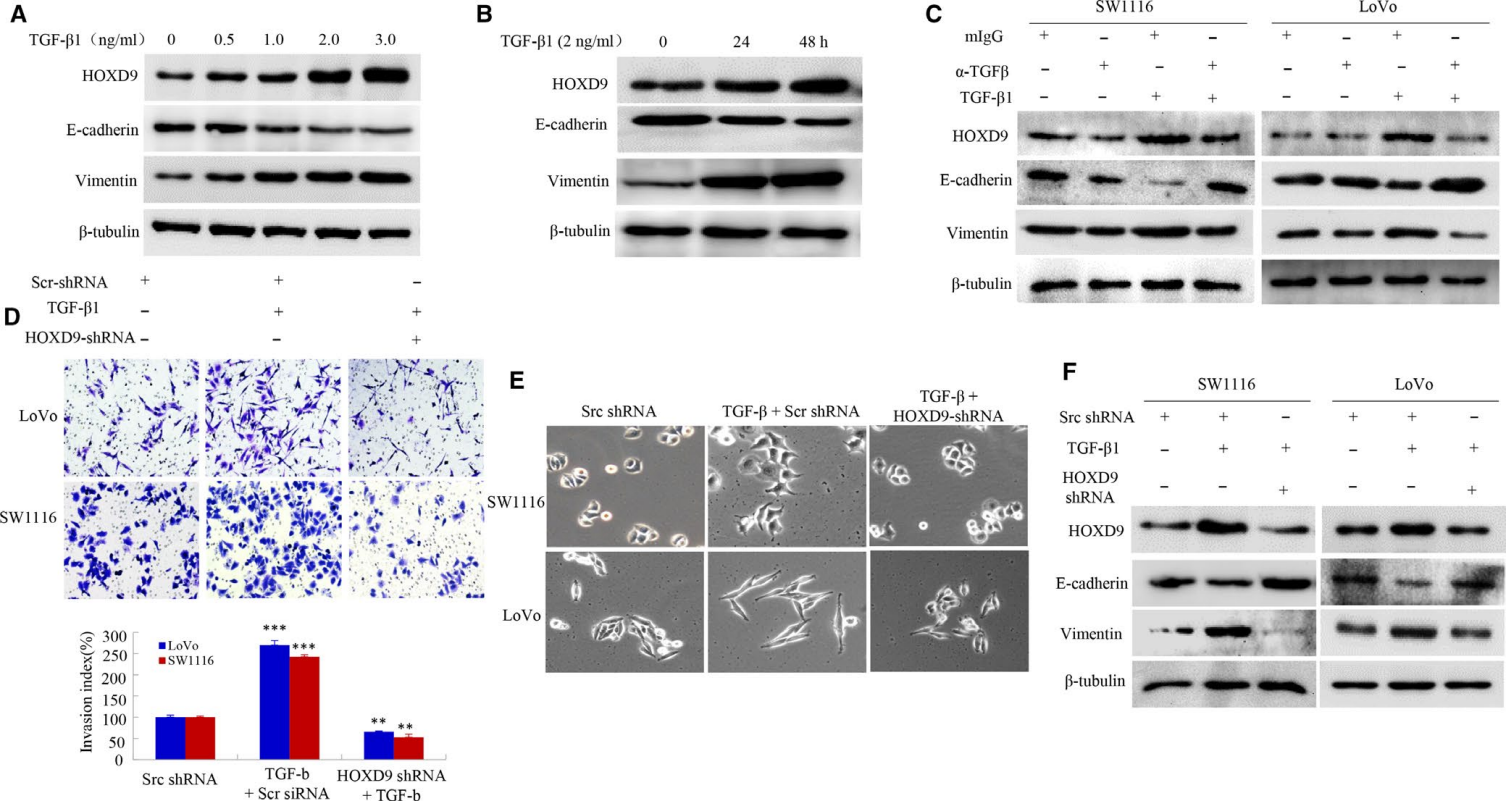
HOXD9 gene is essential for TGF‐β1 ‐induced EMT and migration of cells in colorectal cancer (CRC). A and B, Expression of HOXD9 in LoVo cells was enhanced depending on the dose after treatment with TGF‐1 as observed in Western blot. C, Treatment of CRC cells with recombinant TGF‐1 (2 ng/mL) together with neutralizing mouse IgG (mIgG) or anti‐TGF‐1 antibody (a‐TGF‐β, 2 mg/mL) for 48 h. D, Transfection of cells of CRC with src shRNA or HOXD9 shRNA for 24 h then with TGF‐1 treatment (5 ng/mL) for 24 h. Images and data of a transwell assay for LoVo and SW1116 cells. ***P < .001, compared to that of SW1116 cells and in the presence of TGF‐β1 in LoVo. **P < .01, compared to cells treated with TGF‐1 and transfected with HOXD9 shRNA (E) Morphology of LoVo and SW1116 cells was visualized using an inverted microscope. F, Whole‐cell extracts were analyzed by western blot using the indicated antibodies. Scale bars represent 50 μm in E

Several lines of evidence suggest that TGF‐β1 contributes to tumor progression by promoting EMT.^17^, ^18^, ^19^, ^20^ Therefore, we tested whether TGF‐β‐ induced EMT affects HOXD9 expression in CRC. First, results revealed that TGF‐β1 treatment significantly enhanced the quantity of HOXD9 at the protein level in CRC cells, an effect that varied with the time and dose. In addition, the treatment with TGF‐β1 increased the levels of vimentin, and decreased E‐cadherin levels, (Figure 5A and B). Second, we showed that inhibition of TGF‐β1 using a neutralizing antibody markedly reduced endogenous HOXD9 levels and TGF‐β‐triggered HOXD9 expression in CRC (Figure 5C).

Thirdly, we examined whether knockdown of HOXD9 by siRNA interference prevents TGF‐β‐induced invasion. As shown in 5D, the TGF‐β‐treated cells promoted cell invasion by approximately 2‐fold compared to the control cells. In contrast, the repression of HOXD9 in CRC cells reversed invasive ability stimulated by TGF‐β. Consistently, the HOXD9 knockdown resulted in altered morphological changes (Figure 5E). Also, the repression of HOXD9 decreased the level of vimentin, whereas it increased the quantity of E‐cadherin, revealed by Western bolt assay (Figure 5F).

Taken together, these data demonstrated that HOXD9 is involved in TGF‐β1‐induced EMT.

### 3.5 HOXD9 is essential for the metastasis of CRC in vivo

**FIGURE 6:**
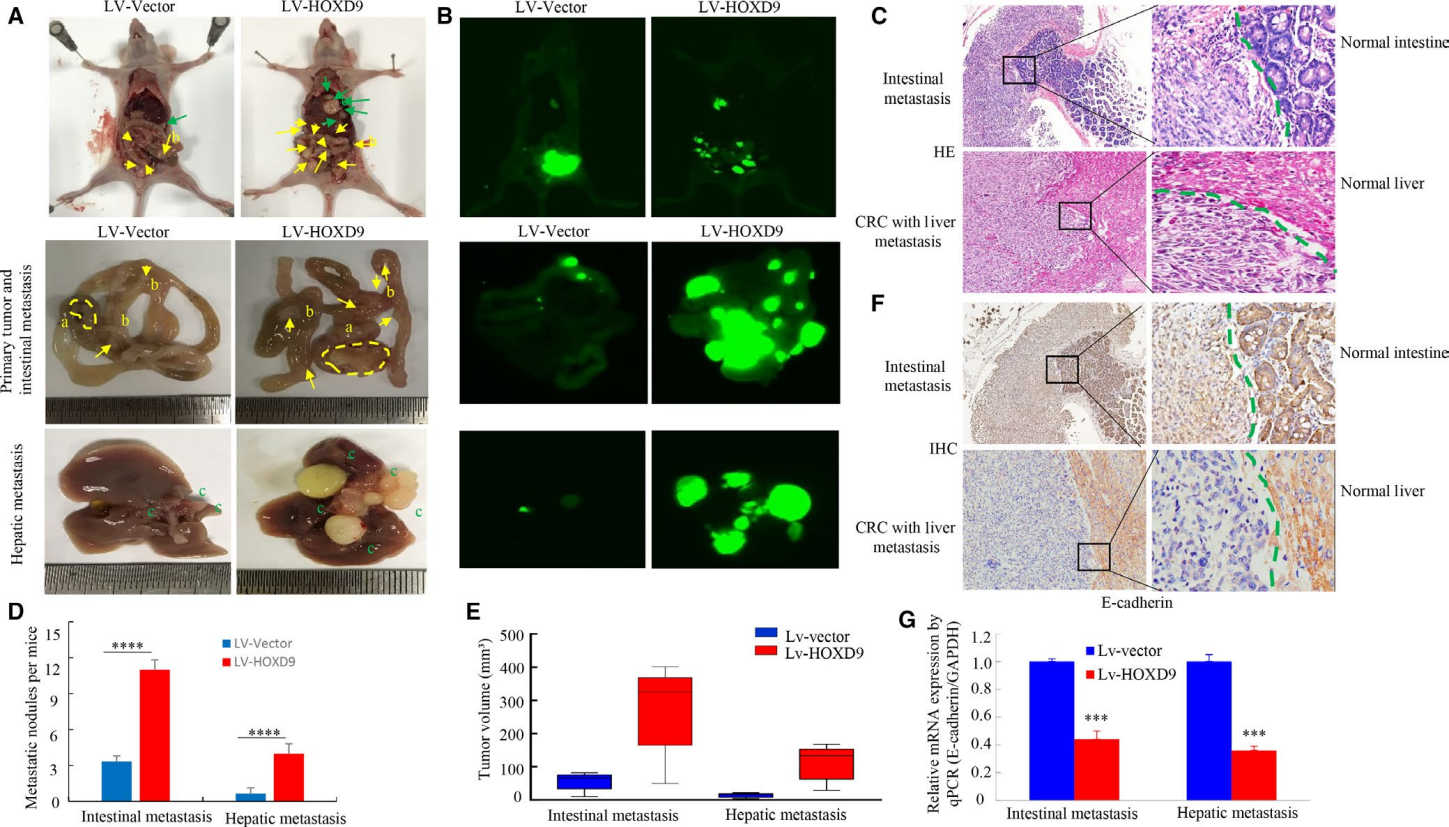
HOXD9 is essential for the metastasis of colorectal cancer in vivo. A, White‐light and B, whole‐body fluorescence images of orthotopic tumors arising from hepatic and intestinal metastases of mice. Yellow arbitrary polygon (a) shows a primary tumor. Yellow (b) and green (c) arrows indicate intestinal and hepatic metastatic nodules. C, The LoVo cells with liver and intestinal cancer tissues were stained using H&E. D, Numbers and E, tumor volumes of metastatic intestinal and hepatic metastases per group were analyzed. The quantity of metastatic nodules in each mouse was determined using a microscope. ****P < .001. F, E‐cadherin expression in the intestine and liver metastasis of LoVo cells was detected by IHC. G, The E‐cadherin expression in tumors derived from LoVo cells was evaluated using a quantitative PCR (qPCR) assay. ***P < .01. Scale bars represent 100 μm in C and F

To determine the effect of HOXD9 in vivo, LoVo cells which expressed lentivirus LV‐HOXD9 and (LV)‐vector, were implanted into the cecum terminus, and organs were subsequently scanned for metastasis by a visualization system. Metastatic lesions were present in the intestine and liver. Larger intestinal or hepatic metastatic nodules were discovered in LV‐HOXD9 groups compared to the LV‐vector group (Figure 6A and B). Histological examination confirmed the existence of CRC metastases into the liver and intestine tissues (Figure 6C). Mice treated with LV‐HOXD9 showed a higher number of hepatic or intestinal metastatic lesions relative to those treated with LV‐vector cells (Figure 6D). Also, the intestinal or hepatic metastatic tumor volumes in the LV‐HOXD9 group were significantly larger than those in the vector group (Figure 6E).

To test HOXD9 contributes to EMT in CRC in vivo, orthotopic xenograft tumors were evaluated by IHC and qPCR. E‐cadherin protein levels were suppressed in cancer tissues in comparison to that in adjacent normal intestinal and liver (Figure 6F). Furthermore, HOXD9 upregulation caused a reduction in E‐cadherin in cancer tissues by q‐PCR assay (Figure 6G).

Taken together, these findings suggested that the HOXD9 may be involved in cell invasion, metastasis in vivo by inducing EMT.

## 4 DISCUSSION

The HOXD9 encourages proliferation and invasion/ migration of cervical, esophageal, glioma, liver, and tumor cells. But, the primary function of HOXD9 in CRC remains a mystery. The current study indicated the vital task of HOXD9 in CRC. Firstly, HOXD9 was upregulated in CRC patients and these were significantly associated with poor clinical outcomes. Secondly, HOXD9 was able to regulate cell cycle progression in CRC cells. Thirdly, HOXD9 encouraged migration of cells including invasion with improved EMT. Fourthly, the expression of HOXD9 was fueled by transforming growth factor (TGF)‐β1. Finally, HOXD9 boosted the spread of tumors through in vivo orthotopic implantation. These results suggested that HOXD9 has a crucial role in metastasis and the development of CRC.

In malignant tumors, aberrant expression of homeobox (HOX) genes is frequently found. Lately, the function of homeobox genes in tumor growth and proliferation has been backed by several studies that have reported that many homeobox genes influence the tumors especially those of the digestive system.^21^, ^22^, ^23^, ^24^ Knight et al demonstrated by RNA‐seq analysis that expression of HOXD9 in normal‐appearing mucosa is significantly higher in the sigmoid colon compared to the rectum.^25^ But, the expression of HOXD9 in CRC is yet to be studied. It was demonstrated in this study that there is a significant relationship between high expression of HOXD9 and CRC. Also, Tabuse et al showed that HOXD9 is highly expressed more in gliomas.^26^ Additionally, the current study showed that overexpression of HOXD9 in tissues of CRC was linked with differentiation, lymph node metastasis, extra serious invasion, and AJCC stage. A new investigation established that HOXA1 which is a homeobox (HOX) gene, is highly expressed in oral squamous cell carcinomas. Its expression is associated with poor prognosis.^27^ The overall survival rate of patients with higher expression of HOXD9 was shorter compared to those with lower expression of the gene, suggesting that the HOXD9 gene presents an independent prognostic aspect for CRC patients. It's crucial to note that the repression of HOXD9 resulted in the significant arrest of G1/G0, with an associated decrease of CDK4/CDK6 expression was evident. Thus, HOXD9 may be involved in cellular proliferation and regulation of the cell cycle.

The function of the HOXD9 gene in regulating tumor growth and metastasis has attracted limited attention. Lv et al were able to demonstrate that high expression of HOXD9 is highly correlated with the metastatic incursion of HCC.^11^ The results of the present study agree with the investigation, suggesting that HOXD9 has a strong influence on the movement and incursion of in vitro tumor cells. Furthermore, overexpression of HOXD9 facilitated by actin depolymerization reduced the E‐cadherin expression and at the same time, improved vimentin (a mesenchymal marker) expression. Ectopic expression of HOXD9 in cells caused functional and molecular characteristics of EMT which exhibited scattering of cells. This phenomenon agreed with the earlier theory that EMT is vital for cancer cells to spread from adjacent tissues and form new tumors in remote areas of the body.^28^, ^29^, ^30^ These findings suggested that HOXD9 influences the invasion and migration of cells through the regulation of EMT. To further understand upstream regulatory elements controlling HOXD9 expression, we analyzed the HOXD9 promoter (>2000 bp) using the jaspar software (http://jaspar.genereg.net/) and found several binding sequence transcription factors involved in EMT. Five potential hif‐1a‐binding sites, seven potential twist1 ‐binding sites, one potential slug ‐binding sites and ten potential snail‐binding sites were found in the HOXD9 promoter. Further studies are needed on the transcriptional regulatory mechanism of the HOXD9 gene in GC cells.

The signaling pathway of TGF‐β has essential roles in various developmental processes and the pathogenesis of several diseases, such as cancer.^20^, ^31^ The TGF‐β1 is able to either hinder or encourage the growth of tumors, and this depends on the direction of tumor progression. During the early stages of tumorigenesis, TGF‐β1 signaling hampers the proliferation of tumors by inducing arrest of cell cycle and programmed cell death.^32^, ^33^ However, at later stages of cancer, it encourages tumor invasion and spread via EMT. Many members of the homeobox gene family have recently been shown to help in facilitating TGF‐β‐induced EMT. Dlx‐2 has also been shown to be involved in TGF‐β and Wnt‐induced EMT in cancers.^34^ Also, HOXD3 supports the progression of lung cancer to a certain extent through TGF‐β‐induced EMT.^17^ Among the HOX proteins, TGF‐β1 induction of EMT in CRC cells was correlated with a significant rise in expression of HOXD9. When the expression of HOXD9 was silenced, TGF‐β1 induction of the EMT phenotype was abolished, and the expression of HOXD9 was also reduced. These findings suggested that HOXD9 could serve as a co‐stimulator in TGF‐β1‐induced EMT in CRC.

In conclusion, the current study suggests that HOXD9 is overexpressed in CRC and increases tumorigenicity and tumor proliferation. Also, ectopic HOXD9 expression influences EMT and tumor spread both in vivo and in vitro. Therefore, the HOXD9 gene could act as an assuring therapeutic approach for treating patients suffering from CRC.

## CONFLICT OF INTEREST

The authors declare no conflict of interest.

## AUTHORS’ CONTRIBUTIONS

This study was designed and conceived by Jide Wang, Jing Xiong, and Li Xiang. Experiments in vitro were performed by Mengwei Liu, Jiayi Li, Yizhi Xiao, Ying Peng, Weiyu Dai. Experiments in vivo were conducted by Linjie Hong, Jing Wang, and Yaying Chen. Wenjing Zhang, Weimei Tang, and Xiaosheng Wu helped with data analysis. Zhizhao Lin, Jianjiao Lin, and Qiong Yang were responsible for the collection of specimens. Yang Bai and Yusi Wang contributed to technical support. Jing Xiong, Side Liu supervised the project and Jide Wang wrote the manuscript. Li Xiang revised the manuscript.

## Data Availability

All remaining data are available within the article and supplementary files, or available from the authors upon request.

## References

[1] Fenton HM , Taylor JC , Lodge JPA , et al. Variation in the use of resection for colorectal cancer liver metastases. Ann Surg. 2019;270(5):892‐898. 31567507 10.1097/SLA.0000000000003534 PMC6867670

[2] Su J , Huang Y‐H , Cui X , et al. Homeobox oncogene activation by pan‐cancer DNA hypermethylation. Genome Biol. 2018;19(1):108. 30097071 10.1186/s13059-018-1492-3 PMC6085761

[3] Xu HM , Zhao HL , Yu J . HOXB5 promotes retinoblastoma cell migration and invasion via ERK1/2 pathway‐mediated MMPs production. Am J Transl Res. 2018;10(6):1703‐1712. 30018711 PMC6038074

[4] Tomotsune D , Shoji H , Wakamatsu Y , Kondoh H , Takahashi N . A mouse homologue of the Drosophila tumour‐suppressor gene l(2)gl controlled by Hox‐C8 in vivo. Nature. 1993;365(6441):69‐72. 8103190 10.1038/365069a0

[5] Hong CS , Jeong O , Piao Z , et al. HOXB5 induces invasion and migration through direct transcriptional up‐regulation of beta‐catenin in human gastric carcinoma. Biochem J. 2015;472(3):393‐403. 26467157 10.1042/BJ20150213

[6] Shen J , Wu H , Gudas LJ . Molecular cloning and analysis of a group of genes differentially expressed in cells which overexpress the Hoxa‐1 homeobox gene. Exper Cell Res. 2000;259(1):274‐283. 10942599 10.1006/excr.2000.4963

[7] Caré A , Silvani A , Meccia E , et al. HOXB7 constitutively activates basic fibroblast growth factor in melanomas. Mol Cell Biol. 1996;16(9):4842‐4851. 8756643 10.1128/mcb.16.9.4842 PMC231486

[8] Axlund SD , Lambert JR , Nordeen SK . HOXC8 inhibits androgen receptor signaling in human prostate cancer cells by inhibiting SRC‐3 recruitment to direct androgen target genes. Mol Cancer Res. 2010;8(12):1643‐1655. 21047772 10.1158/1541-7786.MCR-10-0111

[9] Svoboda LK , Harris A , Bailey NJ , et al. Overexpression of HOX genes is prevalent in Ewing sarcoma and is associated with altered epigenetic regulation of developmental transcription programs. Epigenetics. 2014;9(12):1613‐1625. 25625846 10.4161/15592294.2014.988048 PMC4622732

[10] Liu DB , Gu ZD , Cao XZ , Liu H , Li JY . Immunocytochemical detection of HoxD9 and Pbx1 homeodomain protein expression in Chinese esophageal squamous cell carcinomas. World J Gastroenterol. 2005;11(10):1562‐1566. 15770739 10.3748/wjg.v11.i10.1562 PMC4305705

[11] Lv X , Li L , Lv LI , et al. HOXD9 promotes epithelial‐mesenchymal transition and cancer metastasis by ZEB1 regulation in hepatocellular carcinoma. J Exp Clin Cancer Res. 2015;34:133. 26514226 10.1186/s13046-015-0245-3 PMC4625617

[12] Zhu H , Dai W , Li J , et al. HOXD9 promotes the growth, invasion and metastasis of gastric cancer cells by transcriptional activation of RUFY3. J Exp Clin Cancer Res. 2019;38(1):412. 31547840 10.1186/s13046-019-1399-1 PMC6755711

[13] Huang X , Xiang LI , Li Y , et al. Snail/FOXK1/Cyr61 signaling axis regulates the epithelial‐mesenchymal transition and metastasis in colorectal cancer. Cell Physiol Biochem. 2018;47(2):590‐603. 29794466 10.1159/000490015

[14] Liang LI , Li X , Zhang X , et al. MicroRNA‐137, an HMGA1 target, suppresses colorectal cancer cell invasion and metastasis in mice by directly targeting FMNL2. Gastroenterology. 2013;144(3):624‐635. 23201162 10.1053/j.gastro.2012.11.033

[15] Thiery JP , Acloque H , Huang RY , Nieto MA . Epithelial‐mesenchymal transitions in development and disease. Cell. 2009;139(5):871‐890. 19945376 10.1016/j.cell.2009.11.007

[16] Yao Y , Gu X , Liu H , et al. Metadherin regulates proliferation and metastasis via actin cytoskeletal remodelling in non‐small cell lung cancer. Brit J Cancer. 2014;111(2):355‐364. 24918821 10.1038/bjc.2014.267 PMC4102939

[17] Miyazaki YJ , Hamada J , Tada M , et al. HOXD3 enhances motility and invasiveness through the TGF‐beta‐dependent and ‐independent pathways in A549 cells. Oncogene. 2002;21(5):798‐808. 11850808 10.1038/sj.onc.1205126

[18] Morrison CD , Parvani JG , Schiemann WP . The relevance of the TGF‐β Paradox to EMT‐MET programs. Cancer Lett. 2013;341:30‐40. 23474494 10.1016/j.canlet.2013.02.048 PMC3752409

[19] Wendt MK , Tian M , Schiemann WP . Deconstructing the mechanisms and consequences of TGF‐β‐induced EMT during cancerprogression. Cell Tissue Res. 2012;347:85‐101. 21691718 10.1007/s00441-011-1199-1 PMC3723118

[20] Han G , Lu SL , Li AG , et al. Distinct mechanisms of TGF‐beta1‐mediated epithelial‐to‐mesenchymal transition and metastasis during skin carcinogenesis. J Clin Invest. 2005;115:1714‐1723. 15937546 10.1172/JCI24399 PMC1142114

[21] Sakai H , Eishi Y , Li XL , et al. PDX1 homeobox protein expression in pseudopyloric glands and gastric carcinomas. Gut. 2004;53(3):323‐330. 14960508 10.1136/gut.2003.026609 PMC1773956

[22] Vider B‐Z , Zimber A , Chastre E , et al. Deregulated expression of homeobox‐containing genes, HOXB6, B8, C8, C9, and Cdx‐1, in human colon cancer cell lines. Biochem Biophys Res Comm. 2000;272(2):513‐518. 10833444 10.1006/bbrc.2000.2804

[23] Liao WT , Jiang D , Yuan J , et al. HOXB7 as a prognostic factor and mediator of colorectal cancer progression. Clin Cancer Res. 2011;17(11):3569‐3578. 21474578 10.1158/1078-0432.CCR-10-2533

[24] Li D , Bai Y , Feng Z , et al. Study of promoter methylation patterns of HOXA2, HOXA5, and HOXA6 and its clinicopathological characteristics in colorectal cancer. Front Oncol. 2019;9:394. 31165042 10.3389/fonc.2019.00394 PMC6536611

[25] Knight JM , Kim E , Ivanov I , et al. Comprehensive site‐specific whole genome profiling of stromal and epithelial colonic gene signatures in human sigmoid colon and rectal tissue. Physiol Genom. 2016;48(9):651‐659. 10.1152/physiolgenomics.00023.2016 PMC5111881 27401218

[26] Tabuse M , Ohta S , Ohashi Y , et al. Functional analysis of HOXD9 in human gliomas and glioma cancer stem cells. Mol Cancer. 2011;10:60. 21600039 10.1186/1476-4598-10-60 PMC3118386

[27] Bitu CC , Destro MFdSS , Carrera M , et al. HOXA1 is overexpressed in oral squamous cell carcinomas and its expression is correlated with poor prognosis. BMC cancer. 2012;12:146. 22498108 10.1186/1471-2407-12-146 PMC3351375

[28] Louie E , Chen XF , Coomes A , Ji K , Tsirka S , Chen EI . Neurotrophin‐3 modulates breast cancer cells and the microenvironment to promote the growth of breast cancer brain metastasis. Oncogene. 2013;32(35):4064‐4077. 23001042 10.1038/onc.2012.417 PMC3998718

[29] Bates RC . Colorectal cancer progression: integrin alphavbeta6 and the epithelial‐mesenchymal transition (EMT). Cell Cycle. 2005;4(10):1350‐1352. 16123591 10.4161/cc.4.10.2053

[30] Rodriguez JA , Huerta‐Yepez S , Law IK , et al. Diminished expression of CRHR2 in human colon cancer promotes tumor growth and EMT via persistent IL‐6/Stat3 signaling. Cell Mol Gastroenterol Hepatol. 2015;1(6):610‐630. 26495412 10.1016/j.jcmgh.2015.08.001 PMC4610032

[31] Xie R , Wang J , Tang W , et al. Rufy3 promotes metastasis through epithelial‐mesenchymal transition in colorectal cancer. Cancer Lett. 2017;390:30‐38. 28089833 10.1016/j.canlet.2017.01.001

[32] Saile B , Matthes N , Knittel T , Ramadori G . Transforming growth factor beta and tumor necrosis factor alpha inhibit both apoptosis and proliferation of activated rat hepatic stellate cells. Hepatology (Baltimore, Md). 1999;30(1):196‐202. 10.1002/hep.510300144 10385656

[33] Lv ZD , Yang ZC , Wang HB , et al. The cytotoxic effect of TGF‐β1 on mesothelial cells via apoptosis in early peritoneal carcinomatosis. Oncol Rep. 2012;27(6):1753‐1758. 22447164 10.3892/or.2012.1735

[34] Lee SY , Jeon HM , Ju MK , et al. Dlx‐2 is implicated in TGF‐beta‐ and Wnt‐induced epithelial‐mesenchymal, glycolytic switch, and mitochondrial repression by Snail activation. Int J Oncol. 2015;46(4):1768‐1780. 25651912 10.3892/ijo.2015.2874

